# Quantitative detection of methylated SOCS-1 in schizophrenia and bipolar disorder considering SOCS-1 -1478 CA/del polymorphism and clinical parameters

**DOI:** 10.1192/j.eurpsy.2022.334

**Published:** 2022-09-01

**Authors:** H.M. Aytac, S. Pehlivan, M. Pehlivan, Y. Oyaci

**Affiliations:** 1 Basaksehir Cam and Sakura City Hospital, Department Of Psychiatry, Istanbul, Turkey; 2 Istanbul Faculty of Medicine, Istanbul University, Department Of Medical Biology, Istanbul, Turkey; 3 Gaziantep University, Department Of Hematology, Gaziantep, Turkey

**Keywords:** bipolar disorder, schizophrénia, SOCS-1, promoter methylation

## Abstract

**Introduction:**

Suppressor of cytokine signaling (SOCS) proteins are the critical negative regulators of immune responses that exert their effects by inhibiting the Jak/Stat signaling pathway.

**Objectives:**

To investigate quantitative detection of methylated SOCS-1 in schizophrenia and bipolar disorder considering SOCS-1 -1478CA/del polymorphism and clinical parameters.

**Methods:**

114 patients with SCZ, 86 patients with BD, and 80 healthy volunteers were included in the case-control study. The patients were consecutively admitted to the outpatient clinic for three months and were evaluated with some scales for clinical parameters. To measure the methylation level of the SOCS-1 gene, bisulfite-converted DNA samples were analyzed using the real-time quantitative methylation-specific PCR method. SOCS-1 -1478CA/del gene polymorphism was evaluated by using the PCR-RFLP.

**Results:**

The SOCS-1 promoter methylation levels of SCZ (p = .001) and BD (p = .024) were found to be significantly different from the control group. SOCS-1 methylation was significantly different between SCZ groups due to the age of onset (p = .009). Again, SOCS-1 methylation was significantly different between BD groups due to YMRS scale scores (p = .027). While the SOCS-1 genotype distributions of SCZ patients were not found to be statistically different from the control group, a significant difference in genotype distribution between BD patients and healthy controls was found (p = .013).

**Conclusions:**

The methylated SOCS-1 quantity in DNA samples of both SCZ and BD patients was significantly lower than in control samples. Whereas the SOCS-1 -1478CA/del polymorphism was not related to SCZ, it may be associated with the BD.

**Disclosure:**

No significant relationships.

